# Supplementary figures and images for: Unregulated GmAGL82 Due to Phosphorus Deficiency Positively Regulates Root Nodule Growth in Soybean
Source: Int J Mol Sci. 2024 Feb 1;25(3):1802. doi: 10.3390/ijms25031802 (PMC10855635; doi:10.3390/ijms25031802)

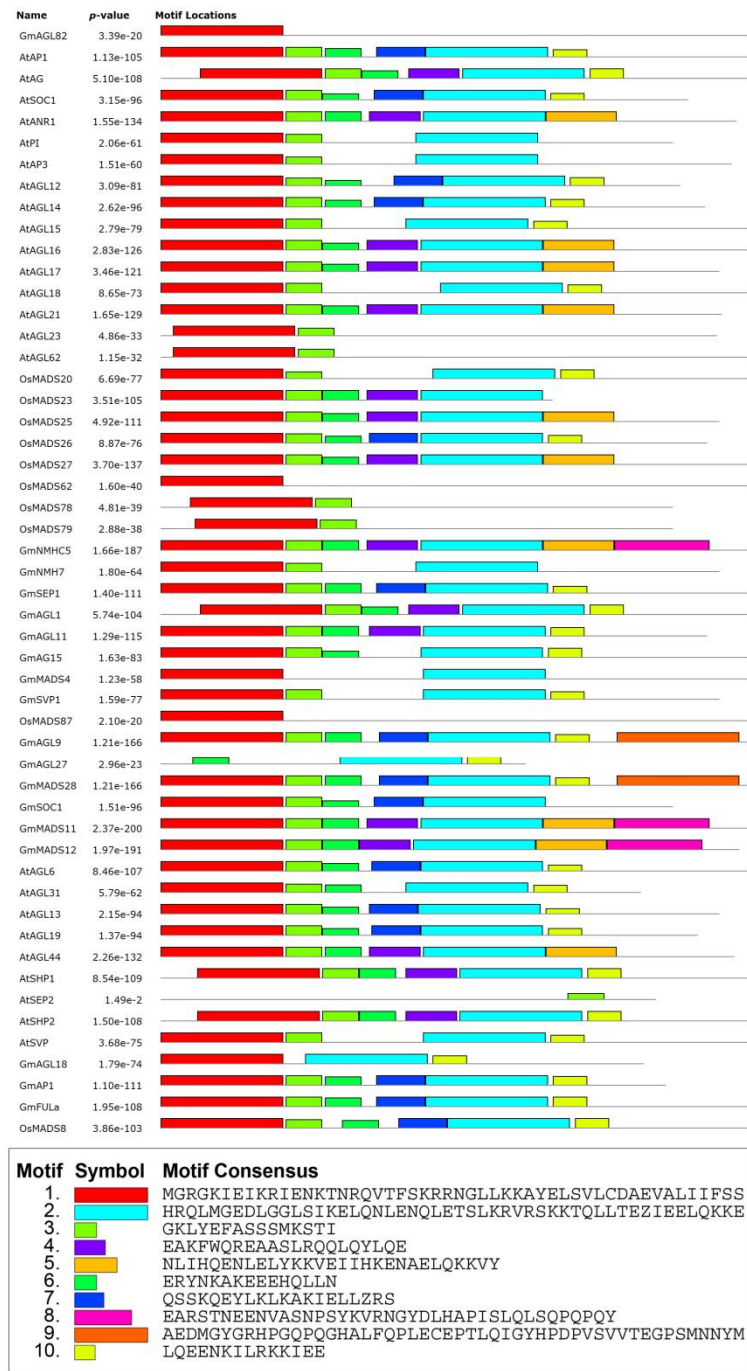

**Figure S1.** Conserved motif analysis of plant MADS proteins.

Supplement: Supplementary file 1 [file ijms-25-01802-s001.zip › Supplementary figures.pdf]
